# Resting Tissue Doppler Imaging for Detecting Coronary Artery Disease in Patients with Preserved Ejection Fraction and No Wall Motion Abnormalities

**DOI:** 10.3390/medicina62081439

**Published:** 2026-07-24

**Authors:** Andrei-Catalin Zavragiu, Petre-Adrian Barzache, Diana-Evelyne Buzzi, Samuel Ardelean, Giulia-Alexandra Bondar, Minodora Andor

**Affiliations:** 1 Doctoral School, “Victor Babes” University of Medicine and Pharmacy, Eftimie Murgu Square No. 2, 300041 Timisoara, Romania; adrian.barzache@umft.ro (P.-A.B.); diana.mailat@umft.ro (D.-E.B.); samuel.ardelean@umft.ro (S.A.); 2 Department of Cardiology, Institute of Cardiovascular Diseases Timisoara, Str. Gheorghe Adam No. 13A, 300310 Timisoara, Romania; bondargiulia30@gmail.com; 3 Medical Semiology II Discipline, Internal Medicine Department, “Victor Babes” University of Medicine and Pharmacy, Eftimie Murgu Square No. 2, 300041 Timisoara, Romania; andor.minodora@umft.ro; 4 Department of Cardiology, ASCAR Clinical Hospital, Bulevardul Revoluției din 1989, No. 12, 300020 Timisoara, Romania

**Keywords:** Tissue Doppler Imaging, coronary artery disease, chronic coronary syndrome, preserved left ventricular ejection fraction, wall motion abnormality

## Abstract

*Background and Objectives*: Coronary artery disease may be difficult to detect by resting echocardiography when left ventricular ejection fraction is preserved and regional wall motion abnormalities are absent. This study aimed to assess whether resting Tissue Doppler Imaging-derived mitral annular velocities can help identify CAD in patients with suspected angina pectoris. *Materials and Methods*: We conducted a cross-sectional observational study of 92 patients hospitalized with suspected angina pectoris who underwent elective coronary angiography at the Institute of Cardiovascular Diseases in Timișoara (January 2025–February 2026). Patients with conditions known to affect TDI-derived parameters were excluded, including previous acute coronary syndrome or myocardial revascularization, significant valvular disease, cardiomyopathies, relevant arrhythmias or conduction abnormalities, permanent pacing, reduced ejection fraction, and pericardial disease. Laboratory and echocardiographic data were collected. ROC curve analysis, univariable logistic regression and multivariable logistic regression were performed to evaluate the diagnostic performance of TDI-derived parameters and their independent association with coronary artery disease. *Results*: Patients with CAD had significantly lower average E′ values (7.4 ± 1.9 vs. 8.9 ± 1.8 cm/s, *p* < 0.001) and average S′ values [7.0 (IQR 6.0–7.5) vs. 9.0 (IQR 8.1–10.0) cm/s, *p* < 0.001], together with higher E/E′ ratios [9.33 (IQR 8.23–11.15) vs. 7.87 (IQR 5.93–9.51), *p* = 0.002]. Average S′ showed the highest discriminative ability for coronary artery disease, with an AUC of 0.899 (95% CI: 0.819–0.952, *p* < 0.0001). The optimal Youden-derived cut-off was ≤7.5 cm/s, yielding 77.42% sensitivity and 93.33% specificity. After adjustment for age, male sex, body mass index, diabetes, smoking status, hypertension and LVEF, dichotomized S′ remained an independent predictor of coronary artery disease (OR = 45.49, 95% CI: 8.03–257.68, *p* < 0.0001), with an adjusted model AUC of 0.92 and 88.04% correct classification. *Conclusions*: TDI, particularly S′ velocity, may be a useful resting echocardiographic parameter for identifying CAD in selected patients with preserved LVEF and no resting regional wall motion abnormalities. Rather than serving as a universal diagnostic marker, S′ should be considered a complementary, easily obtainable parameter that may improve non-invasive assessment in this specific clinical setting.

## 1. Introduction

The role of echocardiography in identifying patients with coronary artery disease remains one of the most underutilized applications of cardiac ultrasound. While its value in diagnosing valvular heart disease, cardiomyopathies, congenital anomalies, and pericardial pathology is widely recognized, its clinical utility in the assessment of CAD is still insufficiently appreciated. In routine clinical practice, the echocardiographic evaluation of chronic coronary syndromes relies primarily on the detection of regional wall motion abnormalities, which can help localize the affected coronary territory; yet, this visual interpretation remains inherently subjective and difficult to quantify [1]. Furthermore, a substantial proportion of patients with significant CAD may present without detectable regional wall motion abnormalities during a resting examination [2].

Stress testing can reproduce anginal symptoms, dyspnea, and ischemic electrocardiographic changes, particularly ST-segment depression [3]. Stress echocardiography has demonstrated good diagnostic accuracy for significant CAD [4,5]. In practice, its use may be limited by false-positive results, inadequate exercise capacity, contraindications to pharmacological stress, and reduced availability in some clinical settings [6,7,8,9]. Although transesophageal echocardiography may provide useful information on proximal coronary anatomy and coronary flow reserve, its routine clinical use is limited by its semi-invasive nature, technical complexity, operator dependence, and restricted assessment of proximal segments [10,11,12]. Speckle-tracking echocardiography has shown diagnostic value in detecting subtle myocardial dysfunction in patients with suspected or established CAD [13,14,15]. Still, its routine use may be limited by the need for dedicated software, post-processing, and specific expertise. Therefore, easily obtainable resting echocardiographic parameters may help complement the diagnostic evaluation.

Tissue Doppler imaging is a valuable echocardiographic technique for assessing both global and regional myocardial systolic and diastolic function [16]. Doppler echocardiography relies on detecting frequency shifts in ultrasound signals reflected from moving structures. While conventional Doppler measures blood flow velocity by analyzing the high-frequency, low-amplitude signals generated by rapidly moving blood cells [17], TDI applies identical physical principles to quantify myocardial motion by capturing the high-amplitude, low-velocity signals of tissue [18].

In CAD and myocardial ischemia, the mitral annular systolic velocity (S′) reflects longitudinal left ventricular systolic function, frequently decreasing early in the ischemic cascade to complement regional wall motion assessment [19]. Furthermore, during diastolic dysfunction, the early diastolic velocity (E′) serves as a relatively load-independent marker of left ventricular relaxation, while the E/E′ ratio is widely utilized to estimate and confirm elevated filling pressures [20,21,22]. Crucially, left ventricular diastolic dysfunction, manifested precisely by reduced E′ velocities and elevated E/E′ ratios, is strongly associated with both the presence and severity of CAD, while also serving as a powerful predictor of adverse cardiovascular outcomes [23,24]. A possible mechanism underlying these changes in tissue Doppler velocities is that myocardial relaxation is an energy-dependent process and is therefore particularly vulnerable to ischemia [25,26]. In addition, recurrent ischemia may induce structural myocardial changes that impair early diastolic and systolic wall motion [27]. Together, these mechanisms may explain why TDI can detect ischemia-related myocardial dysfunction even at rest, before overt systolic impairment or regional wall motion abnormalities become evident.

Although previous studies have demonstrated alterations in TDI-derived parameters in patients with CAD, heterogeneities in study populations and imaging protocols challenge the direct comparison of these findings. Specifically, various concomitant cardiac conditions and resting regional wall motion abnormalities can independently distort mitral annular velocities. Consequently, data focusing strictly on patients with preserved LVEF, an absence of resting wall motion abnormalities, and no confounding clinical pathologies remain highly limited. Addressing this gap, a simplified assessment averaging the septal and lateral mitral annular velocities may offer a practical and easily reproducible approach for routine clinical integration. Therefore, the aim of the present study was to evaluate whether resting TDI-derived parameters, including S′, E′, and the E/E′ ratio, can serve as valuable complementary tools for detecting CAD within a meticulously selected cohort following the exclusion of confounding pathologies known to influence these measurements.

## 2. Materials and Methods

### 2.1. Study Design and Population

We performed a prospective cross-sectional observational study including 92 patients hospitalized at the Institute of Cardiovascular Diseases in Timișoara between January 2025 and February 2026.

Patients included in the study were admitted with typical anginal symptoms for elective coronary angiography. Typical angina was defined according to European Society of Cardiology criteria as chest discomfort, commonly described as pressure, heaviness, or tightness, triggered by physical exertion or emotional stress and relieved within minutes by rest or nitroglycerin [28].

Because TDI-derived parameters (S′, E′, and E/E′) are influenced by several cardiac conditions, we excluded patients with history of acute coronary syndrome or prior myocardial revascularization, including percutaneous coronary intervention with stent implantation or coronary artery bypass grafting, significant valvular heart disease (moderate to severe stenosis or regurgitation), cardiomyopathies (hypertrophic, dilated, restrictive, or infiltrative), clinically relevant arrhythmias (including atrial fibrillation/flutter), conduction abnormalities (right/left bundle branch block) or permanent pacing, heart failure with reduced ejection fraction, and pericardial disease.

The study population was stratified into two groups based on coronary angiography findings. Patients were assigned to the Coronary Disease group if they had at least one coronary stenosis ≥50%, whereas those with coronary stenosis <50% or no angiographic coronary lesions were classified into the No-Coronary Disease group.

An additional exploratory analysis was performed to evaluate the relationship between TDI-derived parameters and the anatomical extent of CAD. For this analysis, patients were categorized into three groups: No-Coronary Disease, single-vessel disease, and multivessel disease, the latter including both two-vessel and three-vessel disease.

Patients’ medical records were systematically reviewed to collect data relevant to cardiovascular risk assessment, including diabetes mellitus, dyslipidemia, hypertension, smoking status, and renal and hepatic function parameters.

**Figure 1:**
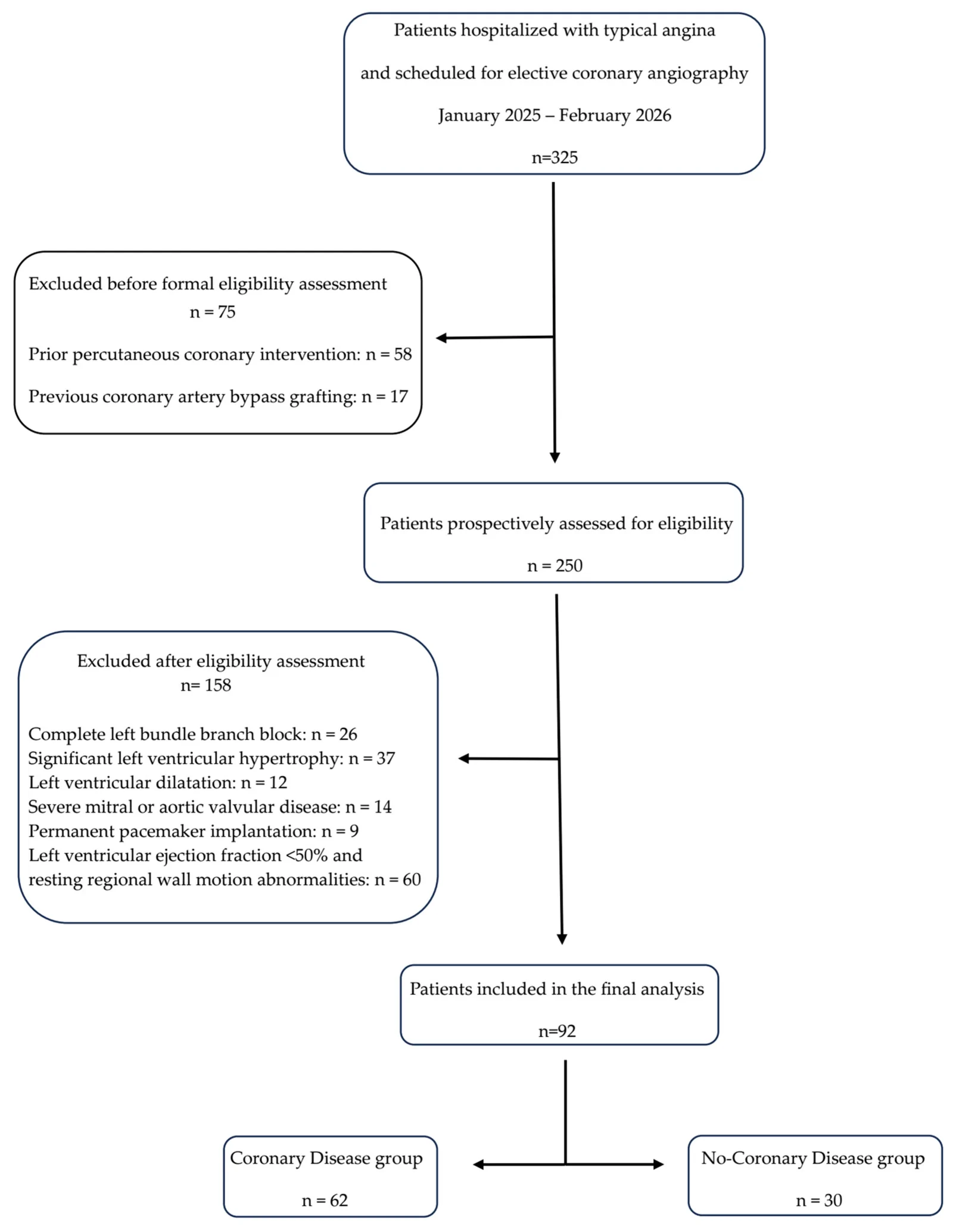
Flowchart.

Among the 325 patients hospitalized with angina, 75 were excluded before formal eligibility assessment because of previous coronary revascularization, including 58 patients with prior percutaneous coronary intervention and 17 patients with previous coronary artery bypass grafting. The remaining 250 patients were prospectively assessed according to the predefined inclusion and exclusion criteria. Of these, 158 were excluded because of conditions known to affect TDI-derived parameters, including complete left bundle branch block, significant left ventricular hypertrophy, left ventricular dilatation, severe mitral or aortic valvular disease, permanent pacemaker implantation, LVEF < 50%, and resting regional wall motion abnormalities. Finally, 92 patients fulfilled all eligibility criteria and were included in the final analysis (Figure 1).

### 2.2. Echocardiographic Examination

All echocardiographic examinations, including TDI acquisition, were performed using a Vivid E95 ultrasound system (GE HealthCare, Horten, Norway) equipped with an M5Sc-D XDclear adult cardiac matrix phased-array transducer operating at 1.4–4.6 MHz. Apical two- and four-chamber views were used to obtain left ventricular end-diastolic and end-systolic volumes by manual endocardial tracing, and LVEF was calculated using the biplane modified Simpson’s method. Left atrial volume was assessed in the apical four-chamber view at end-systole using the area–length method. Diastolic function was evaluated by pulsed-wave Doppler at the mitral valve level, including peak early I and late (A) trans-mitral inflow velocities, with the E/A ratio subsequently calculated. TDI-derived systolic (S′), early diastolic (E′), and late diastolic (A′) myocardial velocities were acquired at the septal and lateral mitral annulus, with three consecutive measurements obtained for each parameter and the mean value used for the final analysis. To minimize respiratory variation and translational artifacts, all acquisitions were performed during quiet respiration at end-expiration. All acquisitions were performed using a standardized echocardiographic protocol and the same machine preset. Imaging depth and sector width were adjusted to optimize temporal resolution for myocardial tissue velocity assessment, while gain, filter, and velocity scale settings were kept consistent across patients. Atrial and ventricular volumes were indexed to the patient’s body surface area. Echocardiographic assessment was performed before coronary angiography, and therefore the echocardiographic measurements were obtained without knowledge of the angiographic findings.

### 2.3. Angiographic Examination

Coronary angiography was performed via radial or femoral access using 5F or 6F Judkins diagnostic and guiding catheters (Cordis Corporation, Miami Lakes, FL, USA), with vascular access obtained using the Seldinger technique. All procedures were jointly performed by two experienced interventional cardiologists. Coronary lesions were evaluated in multiple angiographic projections and, when appropriate, quantitatively assessed using the angiography system’s integrated measurement software, in order to improve the accuracy of stenosis grading and reduce the limitations of visual estimation. Routine functional assessment using fractional flow reserve or instantaneous wave-free ratio was not performed, as pressure-wire-based physiological assessment and the required consumables were not routinely available at the center where coronary angiographies were performed.

### 2.4. Statistical Analyses

Statistical analyses were performed using JASP version 0.19.1 (JASP Team, Amsterdam, The Netherlands) and MedCalc Statistical Software version 23.4.5 (MedCalc Software Ltd., Ostend, Belgium). Normality of distribution was assessed using the Shapiro–Wilk test and homogeneity of variances was evaluated using Levene’s test before performing parametric analyses. Variables with a normal distribution were expressed as mean ± standard deviation and analyzed using parametric tests (Student’s or Welch’s *t*-test). Non-normally distributed variables were expressed as median (interquartile range, 25^th^–75^th^ percentile) and analyzed using nonparametric tests, such as the Mann–Whitney U test for comparisons between groups. The rank-biserial correlation was used as a measure of effect size to quantify the magnitude of the difference between groups. Categorical variables were represented in terms of frequency (N) and percentage (%). Differences between proportions were assessed using the Chi-square test. For the exploratory subgroup analysis according to the anatomical extent of CAD, comparisons of parameters were performed using the Kruskal–Wallis test, given the relatively small subgroup sizes and to ensure consistency across parameters. When the global Kruskal–Wallis test was statistically significant, post hoc pairwise comparisons between subgroups were conducted using Dunn’s test with Holm correction for multiple comparisons. Data from this analysis were reported as median and interquartile range.

Receiver operating characteristic (ROC) curve analysis was performed to evaluate the diagnostic performance of the investigated parameters for identifying the presence of CAD. The area under the ROC curve (AUC) was calculated as a measure of overall discriminative ability. Optimal cut-off values were determined using the Youden index. For each selected cut-off, sensitivity, specificity, and 95% confidence intervals were reported. Binary logistic regression was selected because the dependent variable, CAD, was dichotomous, defined as presence or absence of disease. In addition, S′ was dichotomized using the optimal ROC-derived cut-off value in order to evaluate its clinical usefulness as a categorical diagnostic marker rather than only as a continuous echocardiographic parameter. Therefore, patients were classified according to whether their S′ value was below or above this threshold, and this binary variable was subsequently entered into the logistic regression model. Clinically relevant covariates, including age, male sex, body mass index, diabetes mellitus, smoking status, hypertension, and LVEF, were included in the multivariable model regardless of their significance in univariable analysis, in order to account for potential confounding effects. Model calibration was assessed using the Hosmer–Lemeshow goodness-of-fit test. The discriminative ability of the multivariable logistic regression model was evaluated using the area under the ROC curve derived from the predicted probabilities of the model. A *p* value of less than 0.05 was considered to indicate statistical significance. Given the exploratory nature of this study, a formal sample size calculation was not performed at the study design stage. Accordingly, to assess the adequacy of the available cohort for ROC curve analysis, we performed a subsequent ROC-based sample size assessment. Assuming a clinically relevant AUC of 0.80 for a TDI-derived parameter, a null hypothesis AUC of 0.50, a type I error of 0.05, a statistical power of 80% (type II error of 0.20), and using the observed negative-to-positive case ratio of 0.48, the minimum required sample size was 29 patients, including 19 positive and 10 negative cases. Since the present study included 92 patients, including 62 positive and 30 negative cases, the available sample size exceeded the minimum required sample size for detecting a clinically relevant discriminative ability in ROC analysis.

## 3. Results

### 3.1. Baseline Clinical Characteristics

The study population included 92 patients, with a median age of 64 years (IQR 57–71), predominantly male (76.1%). The baseline characteristics of the study population are presented in Table 1. Cardiovascular risk factors were common, including smoking (44.6%) and diabetes mellitus (27.2%). The median body mass index was 28.6 kg/m^2^ (IQR 25.6–32.2), indicating an overall overweight population. CAD was present in 67% (62) of patients. Among those with CAD, single-vessel disease was observed in 28.3%, two-vessel disease in 23.9%, and three-vessel disease in 15.2% of cases. Left ventricular systolic function was preserved, with a median ejection fraction of 55% (IQR 55–57) and a mean TAPSE of 2.36 cm (±0.37). Diastolic function parameters showed a median E/A ratio of 0.81 (IQR 0.73–1.02) and a median E/E′ ratio of 8.9 (IQR 7.5–10.6), while mean E′ average was 7.9 cm/s (±1.9) and median S′ average was 7.5 cm/s (IQR 6.5–8.6). Laboratory parameters were within expected ranges, including a median creatinine level of 87.5 µmol/L (IQR 77.8–102.5), fasting glucose of 6.22 mmol/L (IQR 5.66–7.1), total cholesterol of 3.83 mmol/L (IQR 3.13–4.55), and LDL cholesterol of 1.97 mmol/L (IQR:1.53–2.53). The study population was stratified into two groups according to the presence of CAD as outlined in Table 2. Patients with CAD were older, although the difference did not reach statistical significance (66 IQR, 57–71 vs. 60 IQR, 53–69 years, *p* = 0.07). A higher proportion of males was observed in the CAD group (82.25% vs. 63.33%, 95%CI: 0.30% to 38.26%, *p* = 0.04). Among cardiovascular risk factors, no significant differences were observed for smoking, hypertension, diabetes mellitus or body mass index. The absence of a significant difference in LDL-C levels between groups may be explained, at least in part, by the fact that most patients were already receiving lipid-lowering therapy, mainly statins, although previous statin treatment and treatment intensity were not systematically quantified in the present study. LVEF was slightly lower in the CAD group (55 IQR, 50–55 vs. 55 IQR, 55–60%, *p* = 0.001), although values remained within the preserved range in both groups. No significant differences were found in TAPSE or left ventricular and atrial volumes. Regarding diastolic function, patients with CAD had lower E′ average values (7.4 ± 1.9 vs. 8.9 ± 1.8 cm/s, 95%CI: 0.36 to 1.26, *p* < 0.001) and higher E/E′ ratios (9.33 IQR, 8.23–11.15 vs. 7.87 IQR, 5.93–9.51, *p* = 0.002). In addition, A-wave velocities were significantly higher in the CAD group (*p* = 0.007), while the E/A ratio did not differ significantly. Importantly, S′ average was significantly lower in patients with CAD (7 IQR, 6–7.5 vs. 9 IQR, 8.1–10 cm/s, *p* < 0.001), indicating impaired longitudinal systolic function. Among laboratory parameters, serum creatinine was significantly higher in the coronary artery disease group (*p* = 0.003), whereas no significant differences were observed for liver enzymes, electrolytes, glucose, or lipid profile.

### 3.2. Exploratory Analysis of TDI-Derived Parameters According to the Anatomical Extent of Coronary Artery Disease

An additional exploratory analysis was performed according to the anatomical extent of CAD, and the results are presented in Table 3 and Table 4. Average S′ velocity differed significantly across the three groups (Kruskal–Wallis statistic = 38.724, *p* < 0.001), with lower values observed in both single-vessel and multivessel disease compared with no CAD. Post hoc Dunn comparisons with Holm correction showed significant differences between no CAD and single-vessel disease (*p* < 0.001), and between no CAD and multivessel disease (*p* < 0.001), while no significant difference was observed between single-vessel and multivessel disease (*p* = 0.885). Average E′ velocity also differed significantly across CAD extent groups (Kruskal–Wallis statistic = 14.415, *p* < 0.001). Post hoc analysis showed significantly lower E′ values in single-vessel disease (*p* = 0.002) and multivessel disease (*p* = 0.004) compared with no CAD, without a significant difference between single- and multivessel disease (*p* = 0.524). Similarly, the E/E′ ratio differed significantly across groups (Kruskal–Wallis statistic = 9.491, *p* = 0.009), with higher values in both single-vessel disease (*p* = 0.018) and multivessel disease (*p* = 0.020) compared with no CAD, while no significant difference was found between single- and multivessel disease (*p* = 0.703). In contrast, A′ velocity did not differ across CAD extent groups (Kruskal–Wallis statistic = 1.391, *p* = 0.499).

### 3.3. Receiver Operating Characteristic (ROC) Curve

**Figure 2:**
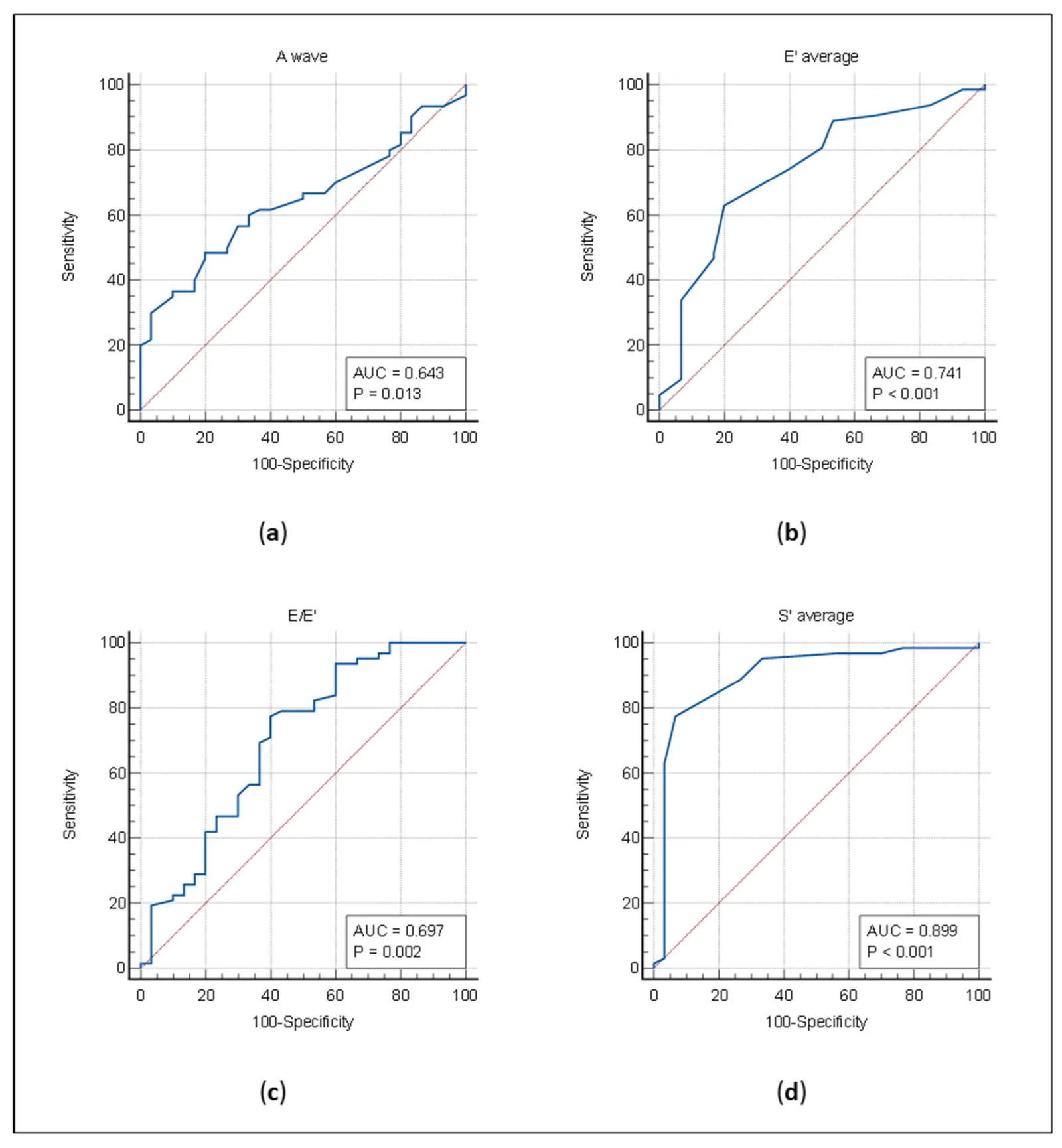
ROC curve analysis of TDI parameters for the detection of CAD. Note. (**a**) ROC curve for transmitral A wave velocity; (**b**) ROC curve for average E′ velocity; (**c**) ROC curve for E/E ratio; (**d**) ROC curve for average S′ velocity.

Comparative ROC analysis demonstrated variable diagnostic performance of echocardiographic parameters for the detection of CAD (Figure 2). Among the evaluated markers, S′ average showed the highest discriminative ability, with an AUC of 0.899 (95% CI: 0.819–0.952, *p* < 0.0001), indicating very good diagnostic accuracy. The optimal cut-off value determined by the Youden index was ≤7.5 cm/s, corresponding to a sensitivity of 77.42% and a specificity of 93.33%. E′ average demonstrated moderate discriminative performance, with an AUC of 0.741 (95% CI: 0.639–0.826, *p* < 0.0001), while the E/E′ ratio showed modest diagnostic capacity (AUC = 0.697, 95% CI: 0.593–0.789, *p* = 0.0016) with an optimal ROC-derived cut-off of >8, corresponding to a sensitivity of 77.42% and a specificity of 60.00%. In contrast, A wave exhibited the lowest discriminative ability, with an AUC of 0.643 (95% CI: 0.535–0.741, *p* = 0.0135), indicating limited diagnostic performance. Overall, S′ average provided the best discrimination for CAD, outperforming both diastolic indices and transmitral flow parameters.

### 3.4. Comparative ROC Analysis with Pairwise Testing

**Figure 3:**
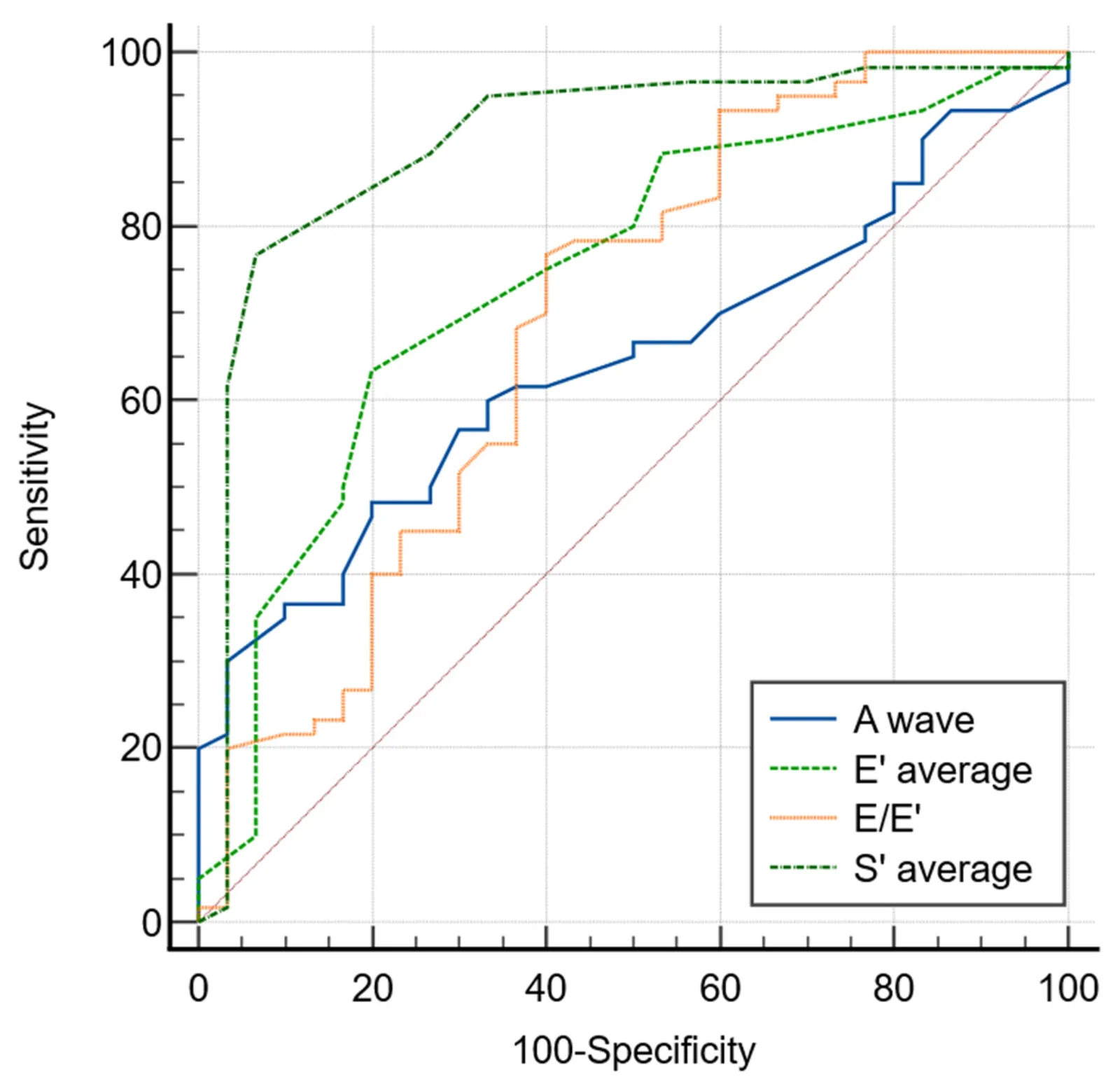
Comparative ROC curve analysis of TDI-derived parameters.

Pairwise comparison of ROC curves using the DeLong method revealed that S′ average had a significantly higher discriminative performance compared to all other evaluated parameters (Figure 3). Specifically, S′ average showed a significantly greater AUC than A wave (ΔAUC = 0.254, 95% CI: 0.115–0.393, *p* = 0.0004), E′ average (ΔAUC = 0.154, 95% CI: 0.026–0.281, *p* = 0.0181), and the E/E′ ratio (ΔAUC = 0.205, 95% CI: 0.063–0.348, *p* = 0.0047).

No statistically significant differences were observed between A wave and E′ average (ΔAUC = 0.100, *p* = 0.188), A wave and E/E′ ratio (ΔAUC = 0.048, *p* = 0.478), or between E′ average and E/E′ ratio (ΔAUC = 0.051, *p* = 0.359), indicating comparable diagnostic performance among these parameters.

### 3.5. Logistic Regression

#### 3.5.1. Univariable Logistic Regression

Dichotomized S′, using the ROC-derived cut-off value of ≤7.5 cm/s, was strongly associated with the presence of CAD. The logistic regression model (Table 5) was statistically significant (χ^2^ = 45.91, *p* < 0.0001), and patients with pathological S′ values had markedly higher odds (Table 6) of CAD (OR = 48.00, 95% CI: 10.16–226.88, *p* < 0.0001). The model correctly classified 82.61% of cases, with a sensitivity of 77.42% and a specificity of 93.33%. ROC analysis of the logistic model showed good diagnostic performance, with an AUC of 0.85 (95% CI: 0.76–0.91) (Table 5).

#### 3.5.2. Multivariable Logistic Regression

Multivariable logistic regression analysis was performed to evaluate whether dichotomized S′ remained independently associated with CAD after adjustment for clinically relevant covariates, including age, male sex, body mass index, diabetes, smoking status, hypertension and LVEF. The overall model (Table 5) was statistically significant (χ^2^ = 54.77, *p* < 0.0001). The Hosmer–Lemeshow goodness-of-fit test did not indicate poor model calibration (χ^2^ = 10.413, *p* = 0.229). Dichotomized S′ remained a strong independent predictor of CAD (OR = 45.49, 95% CI: 8.03–257.68, *p* < 0.0001). In univariable analysis, LVEF was significantly associated with angiographically documented CAD (OR = 0.79, 95% CI: 0.68–0.92, *p* = 0.002), but this association was no longer significant after multivariable adjustment (adjusted OR = 0.81, 95% CI: 0.64–1.03, *p* = 0.09) (Table 6).

The model demonstrated good discriminative performance, with an AUC of 0.92 (95% CI: 0.84–0.96), and correctly classified 88.04% of cases (Table 5).

## 4. Discussion

The present study demonstrates that resting TDI-derived parameters, specifically S′, E′, and the E/E′ ratio, may help identify the presence of CAD in a highly selected population with preserved LVEF and no resting wall motion abnormalities. By excluding confounding cardiac pathologies, our findings underscore the intrinsic value of these resting parameters as a practical, reproducible, and non-invasive complementary tool. This approach unmasks underlying myocardial ischemia in a specific clinical niche where conventional resting echocardiography typically fails to provide diagnostic clues.

Previous studies have shown that both S′ and E′ are highly sensitive to ischemia, with ischemic impairment leading to a reduction in both systolic longitudinal velocity and early diastolic relaxation velocity. In contrast, late diastolic A′ velocity may increase during ischemia, but this response is usually reduced and develops later [29,30]. This may be explained by the fact that systolic myocardial motion and early diastolic relaxation are energy-dependent processes and are therefore particularly sensitive to reduced myocardial oxygen supply [31].

Among the evaluated parameters, average S′ velocity showed the strongest diagnostic performance, with significantly lower values in patients with CAD and the highest discriminative ability on ROC analysis. Importantly, the association between dichotomized S′ and CAD remained significant after adjustment for age, sex, body mass index, diabetes, smoking status, hypertension, and LVEF. These findings suggest that impaired longitudinal systolic function may be detectable at rest even when conventional resting echocardiography does not show overt regional wall motion abnormalities.

Regarding the diastolic indices, E′ showed a numerically higher discriminative performance than the E/E′ ratio for identifying CAD. This may suggest that, in this selected population with preserved LVEF and no resting wall motion abnormalities, impaired early myocardial relaxation may be more directly related to ischemia-related longitudinal dysfunction than the E/E′ ratio, which also depends on transmitral inflow and loading conditions. Nevertheless, the difference between E′ and E/E′ was not statistically significant in pairwise ROC comparison, indicating that both parameters should be interpreted as complementary markers of diastolic involvement rather than as competing diagnostic indices.

These findings align with previous evidence showing that TDI-derived mitral annular velocities provide a quantitative assessment of systolic and diastolic myocardial function and may have diagnostic relevance in coronary artery disease [32].

Similarly, earlier reports have shown reduced S′ and E′ velocities and increased E/E′ values in patients with angiographically confirmed CAD, whereas conventional parameters such as LVEF and E/A did not differ significantly from controls [33,34]. In contrast to the study by Sun et al. [33], our population was specifically restricted to patients with no resting regional wall motion abnormalities, allowing a more focused evaluation of subtle myocardial dysfunction in the absence of overt systolic impairment, similar to the approach reported by Bolognesi et al. [34].

The potential influence of diabetes on myocardial tissue velocities should also be considered. In the study by Dounis et al. [35], patients with CAD, patients with type 2 diabetes mellitus, and controls were analyzed as distinct groups, all with preserved left ventricular ejection fraction. In diabetic patients, the main abnormality involved diastolic function, reflected by reduced early diastolic myocardial velocity (E′), as also described by Marwick [36]. By contrast, patients with CAD showed impairment of both diastolic and systolic longitudinal function, with reduced E′ and S′ velocities. In the present study, diabetic patients were included in both the CAD and no-CAD groups, without a statistically significant difference in diabetes prevalence between groups. Therefore, although diabetes may influence diastolic myocardial velocities, the reduction in both E′ and S′ observed in patients with CAD is unlikely to be explained solely by diabetes distribution and appears more closely related to angiographically documented CAD. Moreover, unlike the diabetic group described in the literature, diastolic dysfunction in CAD patients was not accompanied by a consistent compensatory increase in late diastolic annular velocity (A′), but rather by impaired longitudinal systolic function, suggesting different pathophysiological mechanisms.

In the present study, the use of average septal and lateral mitral annular velocities allowed a simplified assessment of longitudinal myocardial function in patients with suspected CAD. This approach was chosen to provide a practical and easily reproducible resting TDI evaluation that could be integrated into routine echocardiographic examinations. At the same time, ischemia-related myocardial dysfunction may involve different ventricular regions depending on the affected coronary territory, and a more extensive multi-site assessment may better capture the spatial heterogeneity of myocardial involvement. In this regard, Hoffmann et al. [37] assessed mitral annular velocities at six annular sites and calculated global mean values, providing a broader evaluation of longitudinal myocardial function. By contrast, our study derived the analyzed parameters from only two annular sites, septal and lateral, which may improve feasibility in daily practice but may offer a less extensive spatial assessment than multi-site TDI. Interestingly, this study also reported a compensatory increase in late diastolic (A′) velocity in one-vessel disease. In contrast, A′ velocity did not differ significantly according to the presence or anatomical extent of CAD in our cohort, suggesting that A′ may be less consistent as a discriminatory marker in resting two-site TDI assessment.

The relationship between diastolic dysfunction and CAD has also been evaluated mainly under exercise or stress conditions. The E/E′ ratio, a marker of increased left ventricular filling pressures, has been reported to be strongly associated with impaired heart rate recovery after exercise, outperforming conventional parameters such as left ventricular ejection fraction and transmitral E/A ratio [38]. Other studies showed that baseline E/E′ values did not differ significantly between patients with and without obstructive CAD, whereas the exercise-induced increase in E/E′ was significantly greater in the CAD group and remained independently associated with obstructive CAD [39]. Exercise TDI has also been proposed as a quantitative adjunct to conventional stress echocardiography, which remains highly dependent on observer experience because visual assessment of wall motion abnormalities is subjective [40]. In contrast to these stress-based investigations, all TDI measurements in the present study were obtained at rest. Despite the absence of stress testing, significant differences in resting E′, E/E′, and S′ were observed between patients with and without CAD. Notably, average E′ showed a numerically higher discriminative ability for angiographically documented CAD than the E/E′ ratio, although the direct comparison between these two parameters did not reach statistical significance. This suggests that impaired early diastolic relaxation may represent a more sensitive resting diastolic marker than estimated filling pressure in this selected population with preserved LVEF and no resting wall motion abnormalities. In addition, transmitral A-wave velocity was significantly higher in patients with CAD, whereas the E/A ratio did not differ significantly between groups. This finding may reflect a greater contribution of late diastolic filling in patients with CAD. Still, its diagnostic performance was limited compared with TDI-derived parameters, supporting the greater value of myocardial tissue velocities over conventional trans-mitral flow indices in this setting.

Recent studies further support the relationship between TDI-derived parameters, coronary disease burden, and advanced deformation imaging. Nabati et al. [41] showed that a higher E/(E′S′) ratio was independently associated with a higher SYNTAX score in patients with NSTEMI, suggesting that combined systolic and diastolic TDI impairment may reflect more extensive coronary atherosclerosis. In contrast, Halvorsrød et al. [42] reported that speckle-tracking-derived strain was superior to automated TDI-based deformation analysis for identifying acute coronary occlusion in patients with suspected NSTEMI, highlighting the added value of advanced deformation imaging in acute ischemic settings. Although both study populations and endpoints differed from ours, these findings support the broader concept that echocardiographic assessment of longitudinal myocardial function may provide clinically relevant information in CAD.

The clinical relevance of TDI-derived parameters is further supported by studies evaluating their prognostic and longitudinal value in cardiovascular disease. Ratneswaren et al. [43] showed that impaired diastolic function assessed by TDI, particularly lower E′ velocity and higher E/E′ ratio, independently predicted long-term cardiovascular mortality over 20 years in hypertensive patients. In another recent study, Yeh et al. [44] reported that pulse-wave TDI-derived time-to-peak velocity was associated with six-month in-stent restenosis after PCI, suggesting that TDI may also reflect persistent abnormalities in myocardial contraction timing after coronary intervention. These findings highlight the need for future prospective studies to determine whether resting TDI-derived parameters, including S′, E′, E/E′, and other timing-related indices, may have prognostic value and could predict clinical outcomes, disease progression, or the need for coronary revascularization in patients with suspected or confirmed CAD.

The present study has several limitations that should be acknowledged. First, the relatively small sample size (n = 92) and the imbalance between the CAD and no-CAD groups may limit the statistical robustness of our findings. These factors likely contributed to the wide confidence intervals and high odds ratios observed in the logistic regression, suggesting limited model stability; thus, these results should be interpreted as evidence of an association rather than a precise risk quantification. Second, the proposed S′ cut-off was dichotomized and evaluated within the same derivation cohort. While clinically practical, this approach may cause information loss, meaning the threshold remains preliminary and requires external validation in larger, multicenter cohorts. Third, the cross-sectional design restricts our conclusions to diagnostic utility rather than prognostic significance, highlighting the need for future longitudinal studies. Finally, there are methodological constraints regarding the echocardiographic assessment: TDI measurements were restricted to two mitral annular sites (septal and lateral) rather than a comprehensive six-site approach, and formal intra- and inter-observer variability analyses were not performed, which limits the evaluation of measurement reproducibility despite the use of a standardized protocol.

## 5. Conclusions

Resting TDI-derived parameters were associated with angiographically documented CAD in a highly selected cohort of patients referred for elective coronary angiography, with preserved LVEF and no resting regional wall motion abnormalities. Average S′ velocity showed the strongest discriminative ability and remained independently associated with CAD after multivariable adjustment. Lower E′ velocity and higher E/E′ ratio were also observed in patients with CAD, suggesting that both longitudinal systolic dysfunction and impaired myocardial relaxation may be detectable at rest in this specific clinical setting. In contrast, A′ velocity did not significantly differ according to the presence or anatomical extent of CAD.

These findings suggest that resting TDI may serve as a simple and widely available complementary echocardiographic approach for the non-invasive assessment of suspected CAD in carefully selected patients. Importantly, the proposed diagnostic performance and S′ cut-off should be considered preliminary and should not be directly extrapolated to broader populations with suspected CAD. External validation in larger, independent, and more heterogeneous cohorts is required before this cut-off can be used as a robust diagnostic threshold.

## Figures and Tables

**Table 1:** Characteristics of study population.

Characteristics	N = 92
Age, years—median (IQR)	64 (57–71)
Male, % (N)	76.08% (70)
Female, % (N)	23.91% (22)
Smoker, % (N)	44.56% (41)
Diabetes mellitus, % (N)	27.17% (25)
Hypertension, % (N)	85.86% (79)
Body mass index, kg/m^2^—median (IQR)	28.55 (25.57–32.21)
Coronary artery disease, % (N)	67% (62)
Single-vessel coronary artery disease, % (N)	28.26% (26)
Two-vessel coronary artery disease, % (N)	23.91% (22)
Three-vessel coronary artery disease, % (N)	15.21% (14)
Left ventricular ejection fraction—median (IQR)	55 (55–57)
TAPSE, cm, mean ± SD	2.36 ± 0.37
End-diastolic volume of left ventricle, mL/m^2^, mean ± SD	56.02 ± 10.3
Left atrium area, cm^2^—median (IQR)	18.8 (16–21)
Left atrium volume, mL/m^2^—median (IQR)	28.73 (22.08–35.78)
E-wave (mitral valve), cm/s—median (IQR)	70 (57.5–80.3)
A-wave (mitral valve), cm/s, mean ± SD	79.7 ± 15.8
E/A ratio—median (IQR)	0.81 (0.73–1.02)
E′ average (mitral annulus), cm/s, mean ± SD	7.9 ± 1.9
E/E′—median (IQR)	8.9 (7.5–10.62)
A′ average (mitral annulus), cm/s—median (IQR)	9.5 (9–11.4)
S′ average (mitral annulus), cm/s—median (IQR)	7.5 (6.5–8.6)
AST, U/L—median (IQR)	24 (21–29)
ALT, U/L—median (IQR)	30 (22.25–38.75)
Creatinine, µmol/L—median (IQR)	87.52 (77.79–102.54)
Natrium, mmol/L—median (IQR)	139 (137–141)
Potassium, mmol/L—median (IQR)	4.1 (3.9–4.47)
Chloride, mmol/L—median (IQR)	103 (102–104)
Fasting blood glucose, mmol/L—median (IQR)	6.22 (5.66–7.1)
Total cholesterol, mmol/L—median (IQR)	3.83 (3.13–4.55)
Low-density lipoprotein, mmol/L—median (IQR)	1.97 (1.53–2.53)
Triglyceride, mmol/L—median (IQR)	1.06 (0.78–1.42)

Note. Data were presented as either % (N), mean ± SD, or median [IQR 25–75%].

**Table 2:** Patient characteristics according to the presence of coronary artery disease.

Characteristics	Coronary Disease N = 62	No-Coronary Disease N = 30	*p* Value	CI (95%)
Age, years—median (IQR)	66 (57–71)	60 (53–69)	0.07	−0.44 to 0.02
Male, % (N)	82.25% (51)	63.33% (19)	0.04	0.30 to 38.26%
Female, % (N)	17.74% (11)	36.66% (11)	0.04	0.30 to 38.26%
Smoker, % (N)	48.38% (30)	36.66% (11)	0.29	−9.75 to 30.87%
Diabetes mellitus, % (N)	32.25% (20)	16.66% (5)	0.11	−4.19 to 31.08%
Hypertension, % (N)	88.7% (55)	80% (24)	0.26	−5.95 to 26.92%
Body mass index, kg/m^2^—median (IQR)	28.85 (26.03–31.88)	27.65 (24.70–33.12	0.46	−0.33 to 0.15
Left ventricular ejection fraction—median (IQR)	55 (50–55)	55 (55–60)	0.001	0.16 to 0.58
TAPSE, cm, mean ± SD	2.34 ± 0.38	2.42 ± 0.35	0.36	−0.23 to 0.63
End-diastolic volume of left ventricle, mL/m^2^, mean ± SD	56.57 ± 9.56	54.89 ± 11.78	0.46	−0.59 to 0.27
Left atrium area, cm^2^—median (IQR)	19.3(16.4–21)	18 (16–22.12)	0.60	−0.29 to 0.19
Left atrium volume, mL/m^2^—median (IQR)	27.66 (22.16–34.44)	29.7 (21.74–37.46)	0.64	−0.19 to 0.30
E-wave (mitral valve), cm/s—median (IQR)	71 (59–80)	65(54–80)	0.52	−0.32 to 0.16
A-wave (mitral valve), cm/s, mean ± SD	82.4 ± 17.2	74.2 ± 10.7	0.007	−1.02 to −0.12
E/A ratio—median (IQR)	0.79 (0.74–0.91)	0.87 (0.72–1.11)	0.2	−0.08 to 0.40
E′ average (mitral annulus), cm/s, mean ± SD	7.4 ± 1.9	8.9 ± 1.8	<0.001	0.36 to 1.26
E/E′—median (IQR)	9.33 (8.23–11.15)	7.87 (5.93–9.51)	0.002	−0.58 to −0.16
A′ average (mitral annulus), cm/s—median (IQR)	9.5 (9–11)	10 (9–11.5)	0.33	−0.12 to 0.36
S′ average (mitral annulus), cm/s—median (IQR)	7 (6–7.5)	9 (8.1–10)	<0.001	0.68 to 0.87
AST, U/L—median (IQR)	25 (21–30)	22 (19–28)	0.33	−0.36 to 0.13
ALT, U/L—median (IQR)	30 (23–39)	29 (22–38)	0.97	−0.24 to 0.25
Creatinine, µmol/L—median (IQR)	92.82 (83.10–106.08)	81.33 (72.49–91.94)	0.003	−0.58 to −0.15
Natrium, mmol/L—median (IQR)	139 (137–141)	140 (138–141)	0.22	−0.09 to 0.39
Potassium, mmol/L—median (IQR)	4.2 (3.9–4.4)	4 (3.9–4.5)	0.45	−0.34 to 0.15
Chloride, mmol/L—median (IQR)	103 (102–104)	103 (102–104)	0.43	−0.15 to 0.34
Fasting blood glucose, mmol/L—median (IQR)	6.28 (5.72–7.27)	6.16 (5.61–6.94)	0.4	−0.35 to 0.14
Total cholesterol, mmol/L—median (IQR)	3.65 (3.00–4.42)	3.88 (3.31–4.65)	0.56	−0.18 to 0.32
Low-density lipoprotein, mmol/L—median (IQR)	1.99 (1.50–2.56)	1.97 (1.66–2.48)	0.95	−0.26 to 0.24
Triglyceride, mmol/L—median (IQR)	1.06 (0.78–1.40)	1.10 (0.77–1.67)	0.86	−0.23 to 0.27

Note. Data were presented as either % (N), mean ± SD, or median [IQR 25–75%].

**Table 3:** TDI-derived parameters according to the anatomical extent of coronary artery disease.

TDI-Derived Parameters	No Coronary Disease N = 30	Single-Vessel Disease N = 26	Multivessel Disease N = 36	*p* Value	CI (95%)
E′ average (mitral annulus), cm/s—median (IQR)	8.7 (8–10)	7 (6–8)	7.5 (6–8.5)	<0.001	0.059 to 0.33
E/E′—median (IQR)	7.87 (5.93–9.51)	9.16 (8.3–11.2)	9.35 (8.13–10.5)	0.009	0.022 to 0.290
A′ average (mitral annulus), cm/s—median (IQR)	10 (9–11.5)	9.5 (9–11)	9.5 (8.3–11)	0.499	0.001 to 0.130
S′ average (mitral annulus), cm/s—median (IQR)	9 (8.1–10)	6.8 (6–7.9)	7 (6.5–7.5)	<0.001	0.311 to 0.577

Note. Data were presented as median [IQR 25–75%].

**Table 4:** Dunn’s post hoc pairwise comparisons of TDI-derived parameters according to CAD extent.

TDI-Derived Parameters	No CAD vs. Single-Vessel Disease	No CAD vs. Multivessel Disease	Single-Vessel vs. Multivessel Disease
E′ average (mitral annulus), cm/s—median (IQR)	*p* = 0.002	*p* = 0.004	*p* = 0.524
E/E′—median (IQR)	*p* = 0.018	*p* = 0.020	*p* = 0.703
A′ average (mitral annulus), cm/s—median (IQR)	*p* = 1	*p* = 0.731	*p* = 1
S′ average (mitral annulus), cm/s—median (IQR)	*p* < 0.001	*p* < 0.001	*p* = 0.885

Note. Data were presented as median [IQR 25–75%].

**Table 5:** Diagnostic performance of univariable and adjusted logistic regression models for CAD.

Model	χ^2^	Model *p* Value	AUC	95% CI	Accuracy	Sensitivity	Specificity
Univariable model: S′ ≤ 7.5 cm/s	45.91	<0.0001	0.85	0.76–0.91	82.61%	77.42%	93.33%
Adjusted model: S′ + clinical covariates	54.77	<0.0001	0.92	0.84–0.96	88.04%	88.71%	86.67%

**Table 6:** Univariable and multivariable logistic regression analysis for predictors of CAD.

Variable	Univariate OR	95% CI	*p* Value	Adjusted OR	95% CI	*p* Value
S′ ≤ 7.5 cm/s	48	10.16–226.88	<0.0001	45.49	8.03–257.68	<0.0001
Age	1.04	0.99–1.10	0.056	1.01	0.94–1.09	0.71
Male sex	2.68	0.99–7.20	0.0507	1.02	0.22–4.67	0.97
BMI	1.01	0.92–1.10	0.78	1.03	0.9–1.18	0.6
Diabetes	2.38	0.79–7.13	0.04	2.89	0.6–13.79	0.18
Smoking status	1.61	0.66–3.95	0.07	1.36	0.25–7.4	0.71
Hypertension	1.96	0.59–6.46	0.27	3.01	0.46–19.67	0.24
LVEF	0.79	0.68–0.92	0.002	0.81	0.64–1.03	0.09

## Data Availability

All data supporting the findings of this study are contained within the article. Additional information can be obtained from the corresponding author upon reasonable request.

## Abbreviations

The following abbreviations are used in this manuscript:

CAD	Coronary artery disease
TDI	Tissue Doppler Imaging
ROC	Receiver operating characteristic
AUC	Area under the ROC curve
LVEF	Left ventricular ejection fraction
GLS	Global longitudinal strain
